# Role of irisin in bone diseases

**DOI:** 10.3389/fendo.2023.1212892

**Published:** 2023-08-04

**Authors:** Ruobing Zhao, Yan Chen, Dongxue Wang, Chunyu Zhang, Henan Song, Guoxin Ni

**Affiliations:** ^1^ School of Sport Medicine and Rehabilitation, Beijing Sport University, Beijing, China; ^2^ Xiyuan Hospital, China Academy of Chinese Medical Sciences, Beijing, China; ^3^ Department of Rehabilitation Medicine, The First Affiliated Hospital of Xiamen University, Xiamen, China

**Keywords:** irisin, bone diseases, osteoarthritis, osteoporosis, rheumatoid arthritis

## Abstract

Bone diseases are common among middle-aged and elderly people, and harm to activities of daily living (ADL) and quality of life (QOL) for patients. It is crucial to search for key regulatory factors associated with the development of bone diseases and explore potential therapeutic targets for bone diseases. Irisin is a novel myokine that has been discovered in recent years. Accumulating evidence indicates that irisin has beneficial effects in the treatment of various diseases such as metabolic, cardiovascular and neurological disorders, especially bone-related diseases. Recent studies had shown that irisin plays the role in various bone diseases such as osteoarthritis, osteoporosis and other bone diseases, suggesting that irisin may be a potential molecule for the prevention and treatment of bone diseases. Therefore, in this review, by consulting the related domestic and international literature of irisin and bone diseases, we summarized the specific regulatory mechanisms of irisin in various bone diseases, and provided a systematic theoretical basis for its application in the diagnosis and treatment of the bone diseases.

## 1 Introduction

Bone diseases arise when normal bone metabolism is disrupted by congenital or acquired factors (1). Common bone diseases include osteoarthritis (OA), osteoporosis (OP), rheumatoid arthritis (RA), etc. These disorders are associated with chronic and recurrent pain due to the abnormal bone metabolism, and the damage gets progressively worse over time, seriously affecting the patient’s quality of life (2). Muscle and bone are both part of the locomotor system and there is a close and inseparable relationship between these two tissues. Recent research has found that muscle and bone coexist and adapt in terms of biosynthesis and metabolism (3). Numerous studies have shown that muscle tissue, as an endocrine organ, secretes myokine which can regulate bone growth and promote or inhibit bone metabolism (4–6). For example, factors such as insulin-like growth factor 1 (IGF-1), fibroblast growth factor 2 (FGF2) and irisin can increase bone mineral density (BMD), while myostatin, interleukin-6 (IL-6) and interleukin-7 (IL-7) will decrease BMD (7). Among them, irisin, a novel myokine that was first identified by Boström et al. in 2012, has been shown that it could be a potential candidate for muscle-osteoblast connectivity, and have a significant correlation with bone health status (8, 9).

It was found that irisin treatment was able to alter the geometry of bones, resulting in longer bones that were more effective in resisting torsional forces. In young male mice, after the injection of vehicle or recombinant irisin (r-irisin) at a low cumulative weekly dose of 100 µg kg (-1), cortical bone density, periosteal perimeter, the index of long bone resistance to torsion and bending strength were increased (10). In another study, a lower dose of r-irisin (1 nM) promotes osteogenic marker gene [alkaline phosphatase (ALP), collagen type 1 alpha-1 (COL1α1)] expressions, ALP activity, and calcium deposition in primary osteoblasts, restoring the reduced osteogenic capacity caused by microgravity (11). Similarly, Colucci et al. also demonstrated that irisin can prevent microgravity-induced impairment of osteoblast differentiation *in vitro* during spaceflight missions (12). In addition, experimental studies by Colaianni et al (13) showed that intermittent administration of irisin in hindlimb-suspended mice prevented the development of both disuse-induced osteoporosis and muscular atrophy. The above studies suggest that irisin may play a key regulatory role in the process of bone remodeling. However, the study of irisin in bone disease is still at a preliminary stage and its potential regulatory mechanisms are not yet clear. Therefore, in this paper, we collated the researches on the treatment of bone diseases by irisin to reveal the specific molecular mechanisms and provide a systematic theoretical basis for the application of irisin in the diagnosis, treatment and research of bone diseases.

## 2 Overview of irisin

Irisin is a polypeptide consisting of 112 amino acids, which is cleaved and secreted by the fibronectin type III domain containing protein 5 (FNDC5). It is highly conserved in all mammalian species sequenced, and mouse and human irisin are 100% identical (8). 72% of circulating irisin is derived from skeletal muscle, and exercise will induce up-regulation of the expression of peroxisome proliferator-activated receptor-γ coactiva-tor-1α (PGC-1α), which in turn increases the expression of FNDC5 in the cell membrane. Then the irisin is secreted into the circulation following proteolytic cleavage from its cellular form, FNDC5. Then the extracellular portion of FNDC5 is sheared to produce irisin and secreted into the circulation (8). In addition to the increased expression of irisin induced by exercise, other factors such as starvation, cold, heat and omega 3 fatty acids can also cause increased secretion of irisin (14–17).

It was found that irisin functions through the αV/β5 integrin to promote osteocyte survival and sclerostin secretion (18). Integrins are transmembrane αβ heterodimers, and at least 18 α and 8 β subunits are known in humans. They are located on the surface of cell membranes and regulate cell-cell and cell-extracellular matrix interactions (19). αV/β5 integrin is a member of the integrin family, which consists of αV subunits and β5 subunits, and has important roles in maintaining capillary integrity, cell adhesion, cell activation, cell migration, cell proliferation and inflammation (20). Kim et al. (18) found in experiments with cultured HEK293T cells that αV/β1 integrin and αV/β5 integrin have significant affinity and response for irisin, and is required for the cellular response to irisin. This study also showed that the antagonistic antibody targeting αV/β5 integrin blocked almost all of the irisin-mediated signaling, downstream gene expression, and expression in osteocytes and fat tissues. This suggests that the pathway involving irisin/αV/β5 integrin may be the primary pathway through which irisin exerts its effects. This is the first identification of an irisin receptor in osteoblasts, and further research is needed to determine if irisin has specificity for other receptors.

Irisin is secreted primarily by skeletal muscle as well as subcutaneous and visceral adipose tissues. However, as showed by immunohistochemical studies, smaller amounts of irisin are also produced by brain, heart, liver, pancreas, spleen, stomach, and testes (21). The main physiological functions of irisin are to promote the conversion of white adipose tissue to brown adipose tissue (22, 23) and to regulate glucose metabolism (24). In addition, there is evidence to suggest that irisin plays a role in inhibiting inflammation and endothelial cell apoptosis, and in reducing neuronal damage (25–27). In recent years, the effects of irisin on the musculoskeletal system have also been widely studied in the scientific community (28).

## 3 Irisin and OA

**Figure 1:**
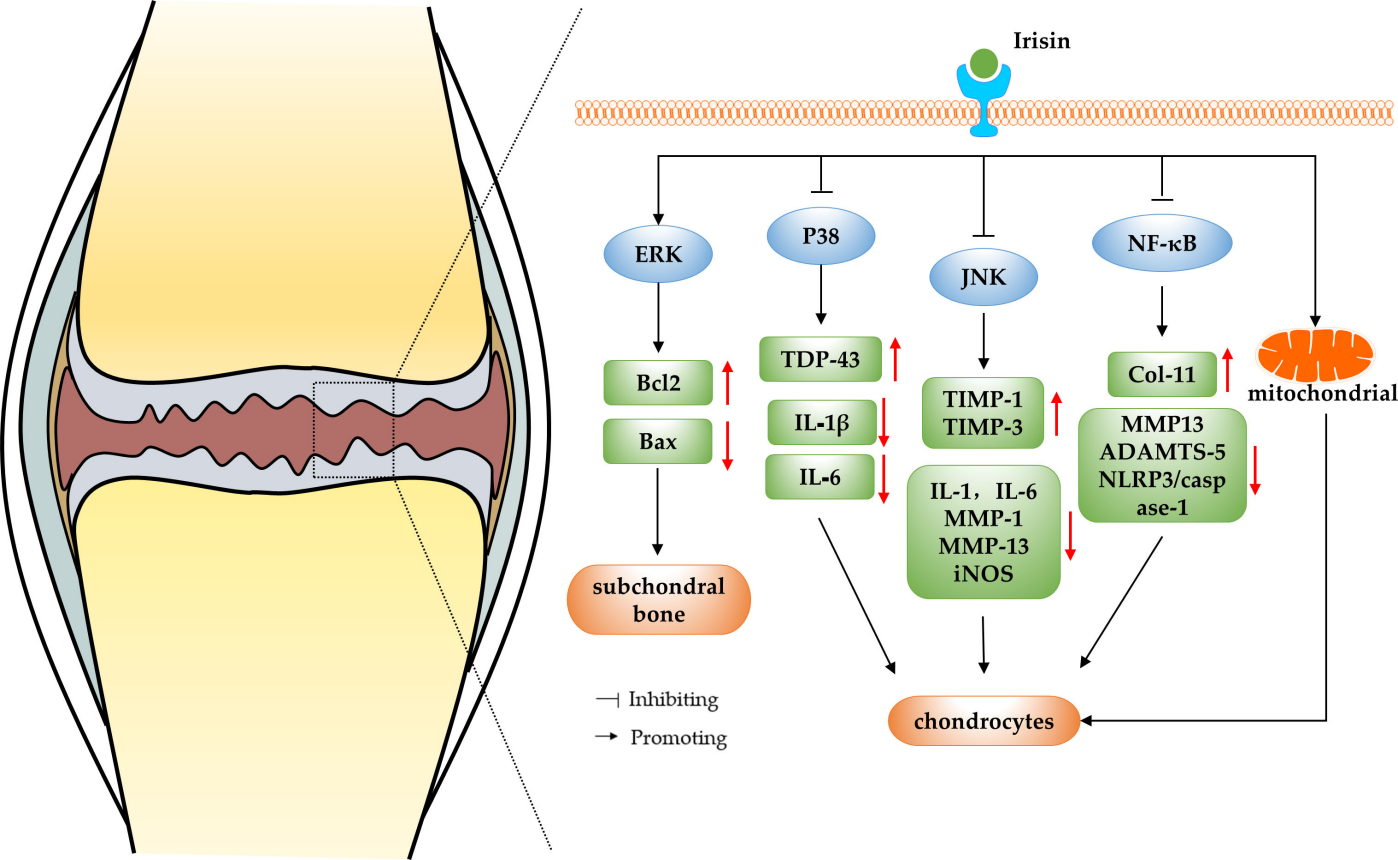
The schematic diagram of irisin regulating OA.

OA is widely accepted as a common degenerative joint disease affected by biomechanics and biochemical signals, which usually results in joint pain, joint stiffness and restricted movement in patients, and in severe cases, even disability (29). Current treatments for OA include mainly surgical and non-surgical treatments, however, these methods only relieve symptoms and do not reverse the progression of OA (30). Irisin is a recently identified myokine, and some researchers found that irisin levels in the serum and synovial fluid (SF) of knee OA patients were negatively correlated with disease severity evaluated by the radiographic Kellgren and Lawrence (KL) grading criteria, suggesting a correlation between irisin and OA (31). In a study conducted by Li et al. (32), cartilage and surrounding tissues were collected from embryonic, newborn, and adolescent mice for immunohistochemical analysis. The findings revealed differential expression patterns of irisin during cartilage development, suggesting its potential role in regulating cartilage development. The immunohistochemical results indicated a decrease in irisin expression in both the cartilage of mice with OA and human OA patients. In addition, they also reported that intra-articular injection of irisin attenuated anterior cruciate ligament transection (ACLT) induced OA progression. Irisin knockout mice developed severe OA while irisin over-expression in both irisin knock in mice and intraarticular injection of irisin protein attenuated OA progression. This study initially demonstrated the potential of irisin to treat and reverse the pathological features of OA. In addition, Posa et al. performed biomolecular analysis and histomorphometry on three-dimensional cultures of human articular chondrocytes that were treated with untagged recombinant irisin. The results demonstrated that irisin has the ability to induce chondrogenic differentiation (33). In recent years, more and more researchers have explored the mechanism of irisin in the regulation of OA. The results suggest that irisin can be involved in regulating chondrocyte metabolism and reducing decreasing apoptosis by regulating MAPK and nuclear factor kappa-light-chain-enhancer of activated B cells (NF-κB) pathways or protecting the mitochondrial function of chondrocytes, thereby slowing down the progression of OA (as shown in **Figure 1**).

### 3.1 Irisin regulate OA by modulating the MAPK pathway

The mitogen-activated protein kinase (MAPK) signaling pathway is found in eukaryotic organisms and includes extracellular regulated kinase (ERK), c-Jun N-terminal kinase (JNK), p38 mitogen-activated protein kinase (p38MAPK) and ERK/BMK1 (34). The MAPK and receptor tyrosine kinases that have been shown to be involved in the pathogenesis of OA are ERK, JNK and p38MAPK (35). Cartilage degradation plays an important role in the pathogenesis of OA, and the MAPK signaling pathway is the main pathway of cartilage degradation, which involves the proliferation, apoptosis, differentiation of chondrocytes and the inflammatory response (36). Therefore, studying the changes and functions of MAPK pathways during articular cartilage degeneration is important to further discover new therapeutic directions for OA and develop new treatment approaches.

ERK, a member of the MAPK family, is a key factor in the transmission of signals from the cell surface to the nucleus, and it plays an important mediating role in OA chondrocyte differentiation and proliferation (37). It has been shown that irisin acts on osteocytes by increasing the expression of the transcription factor activated transcription factor 4 (Atf4) through an Erk-dependent pathway, which in turn acts on osteoblasts, increasing their functions and exerts anti-apoptotic effects. This study revealed underlying mechanisms of irisin action on osteocytes and may encourage research on irisin for the treatment of bone diseases (38). In addition, He et al. (39) randomly divided three-month-old male C57BL/6J mice to groups that underwent sham operation, and ACLT intraperitoneally injected with vehicle or irisin *in vivo*, and injected the mice weekly from the first post-operative day and assessed the microstructure of the subchondral bone after four weeks. The results showed that r-Irisin can alleviate symptoms of OA by activating the ERK pathway *in vitro* to reduce decreasing apoptosis of osteocytes and improving the microarchitecture of subchondral bone. In addition, the p38-MAPK family, which is also a member of the MAPK family like ERK, plays a crucial role in the degradation of OA cartilage. Mechanical stress, cytokines and inflammatory mediators can activate the p38MAPK pathway, thereby inducing the expression of inflammatory mediators such as cyclooxygenase-2 (COX-2), inducible nitric oxide synthase (iNOS) and various MMPs, and promoting the development of OA (40). It was shown that irisin significantly promoted the transcriptional and translational repression of transactive response DNA binding protein 43 (TDP-43) and inhibit phosphorylation of P38 in cartilage tissues of OA model rats. The study also performed validation experiments with the p38MAPK inhibitor SB203580, which showed that inhibition of p38 phosphorylation significantly blocked the effect of irisin in alleviating OA. This further demonstrates that irisin can inhibit inflammatory responses and protect chondrocytes from OA symptoms by inhibiting the activation of the p38MAPK pathway (41). Moreover, the other important isoform of the MAPK pathway——JNK pathway, plays an important role in cell development, apoptosis and stress. Factors such as tumor necrosis factor-α (TNF-α) can activate the JNK pathway, thereby promoting apoptosis or death of chondrocytes and accelerating OA progression (42). Mazur-Bialy et al. (43) investigated the role of irisin in the downstream pathway activation of Toll-like receptor 4 in RAW 264.7 macrophages stimulated with lipopolysaccharide. The results have shown that irisin can exert anti-inflammatory effects by inhibiting the phosphorylation of JNK and ERK, confirming the irisin has been shown to regulate the JNK pathway. Further studies showed that irisin may stimulate human osteoarthritic chondrocytes (hOAC) proliferation and anabolism inhibiting catabolism through p38, protein kinase B (Akt), JNK, and NF-κB inactivation *in vitro*, and attenuate OA-related cartilage degeneration (44).

In summary, p38, JNK and ERK in the MAPK pathway are all inextricably linked to the formation and progression of OA. Irisin can inhibit osteoblast apoptosis by activating the ERK pathway, or inhibit the p38MAPK and JNK pathways to directly target chondrocytes and enhance cellular anabolism and reduce catabolism, thereby attenuating OA progression. The evidence suggests that irisin has a potential positive role in the treatment of OA by modulating the MAPK pathway, but the specific regulatory mechanisms involved have not yet been elucidated and further studies are needed to explore them in depth.

### 3.2 Irisin regulate OA by inhibiting the NF-κB pathway

The NF-κB is a transcription factor that regulates the expression of a wide variety of genes involved in immune and inflammatory responses, cell proliferation, tumorigenesis, cell survival, and development (45). Furthermore, the NF-κB pathway is a key molecular pathway in the process of OA cartilage degradation, which exacerbates OA chondrocyte apoptosis and cartilage inflammatory responses by promoting the secretion of multiple degradative enzymes and the synthesis of catabolic factors (46). Inhibition of the NF-κB pathway can modulate the inflammatory response, thereby reducing the erosion of cartilage and bone tissue and decreasing osteoclast differentiation (47). Therefore, searching for regulators of the NF-κB pathway and modulating NF-κB activity would provide new potential options for the diagnosis and treatment of OA.

Numerous studies have previously reported the regulatory effects of irisin on the NF-κB pathway, for example, irisin reduces the inflammatory response by decreasing the level of NF-kB in serum, thus treating rats with acute pelvic inflammatory disease (APID) (48), irisin exerts anti-inflammatory and anti-apoptotic effects by blocking NF-κB-signal transmission, and plays a protective role against liver injury (49), kidney injury (50) and metabolic diseases (51). The above studies demonstrate the link between irisin and the NF-κB pathway, which is not only reflected in the aforementioned diseases, but also widely responsive to the exploration of OA-related mechanisms. It has been shown that treatment of a SW1353 chondrosarcoma cell line with irisin resulted in inhibition of the Wnt/β-catenin and NF-κB signaling pathways, and irisin exerted a protective effect on SW1353 cells by reducing the expression level of MMP-13 and increasing the expression level of collagen II (Col-II), which demonstrates that irisin may contribute to the treatment of OA (52). In addition, Jia et al. (53) investigated the anti-inflammatory and anti-pyroptosis mechanism of irisin in OA chondrocytes in a rat OA model and showed that irisin not only recovered the expression of collagen II and attenuated that of MMP-13 and a disintegrin and metalloprotease with thrombospondin motif 5 (ADAMTS-5) in interleukin-1 β (IL-1β)-induced OA chondrocytes by inhibiting the PI3K/Akt/NF-κB pathway, but also inhibited the activity of nod-like receptor pro-tein-3 (NLRP3)/caspase-1, thus ameliorating pyroptosis in chondrocytes and improving OA as well as achieving a therapeutic effect.

In summary, irisin can inhibit the NF-κB pathway, thereby suppressing the inflammatory response, protecting chondrocytes and helping to attenuate the pathological progression of OA. Notably, previous studies have generalized that activation of the NF-κB signaling pathway not only leads to articular cartilage damage, but also plays an important role in OA-related pathological changes such as abnormal subchondral bone reconstruction and synovial inflammation (54–56). However, no studies have been conducted to determine whether irisin can act on these OA pathological processes by acting on the NF-κB pathway. In the future, a lot of experimental studies are needed to fill this gap, so as to fully understand the molecular mechanism of NF-κB pathway targeting by irisin and bring new therapeutic strategies and targets for clinical OA treatment.

### 3.3 Irisin regulate OA by protecting the mitochondrial function of chondrocytes

Recent studies have shown that mitochondrial dysfunction plays a key role in the pathophysiology of OA, and it will lead to excessive production of reactive oxidative free radicals, resulting in oxidative damage to protein and DNA stability, thus impeding ECM anabolism in chondrocytes, thereby promoting the development of OA (57, 58). The mitochondrial dysfunction in OA chondrocytes is mainly characterized by reduced activities of the respiratory chain complex and ATP generation, loss of mitochondrial membrane potential, altered mitochondrial biogenesis, and impaired activation of autophagy (59–61). Based on above evidence, it is important to explore potential strategies to treat OA from the mitochondrial pathway. Some researchers have reported that the exercise-increased mitochondrial fission and selective autophagy (mitophagy) in ischemic limbs can be initiated by the PGC1a/FNDC5/irisin pathway (62). Further studies have demonstrated the modulatory effects of irisin on mitochondrial dysfunction, for example, exogenous irisin treatment has been found to inhibit excessive mitochondrial fission, promote mitochondrial biogenesis, reduce oxidative stress, and thus alleviate hepatic I/R injury in mice (63), and irisin abrogates mitochondrial dysfunction, oxidative stress, and apoptosis through Fundc1-related mitophagy in lipopolysaccharide- (LPS-) stimulated H9C2 cardiomyocytes, which is a potentially useful treatment for septic cardiomyopathy (64). Thus, irisin may be able to alleviate OA by protecting chondrocyte mitochondrial function. Wang et al. (65) found that intra-articular administration of irisin further alleviated symptoms of medial meniscus destabilization, like cartilage erosion and synovitis, while improved the gait profiles of the injured legs. *In vitro*, irisin improved IL-1β-mediated growth inhibition, loss of specific cartilage markers and glycosaminoglycan production by chondrocytes. Irisin also reversed sirtuin3 (Sirt3) and uncoupling protein 1 (UCP-1) pathways, thereby improving mitochondrial membrane potential, ATP production, and catalase to attenuated IL-1β-mediated reactive oxygen radical production, mitochondrial fusion, mitophagy, and autophagosome formation. This study reveals the molecular mechanisms by which irisin inhibits mitochondrial dysfunction and oxidative stress in chondrocytes during OA development and demonstrates that irisin impairs apoptosis and ECM in inflammatory chondrocytes by maintaining mitochondrial activities, thus highlights the remedial potential of irisin recombinant protein for OA disease.

In summary, irisin is able to preserve mitochondrial biogenesis, kinetics and autophagy to inhibit inflammation-mediated oxidative stress and insufficient extracellular matrix production, thereby increasing the survival of inflamed chondrocytes and slowing cartilage degeneration and OA progression. The role of irisin in the regulation of mitochondrial function in chondrocytes is unquestionable, but the mechanisms of action of the signalling pathways involved in this process need to be clarified. Furthermore, the pathological process of OA does not only involve chondrocytes, and whether the mitochondrial function-enhancing effect of irisin applies to other cell types, including osteoblasts and osteoclasts in subchondral bone, needs to be further investigated as a key to assessing the efficacy of irisin in targeting OA via the mitochondrial pathway.

## 4 Irisin and OP

**Figure 2:**
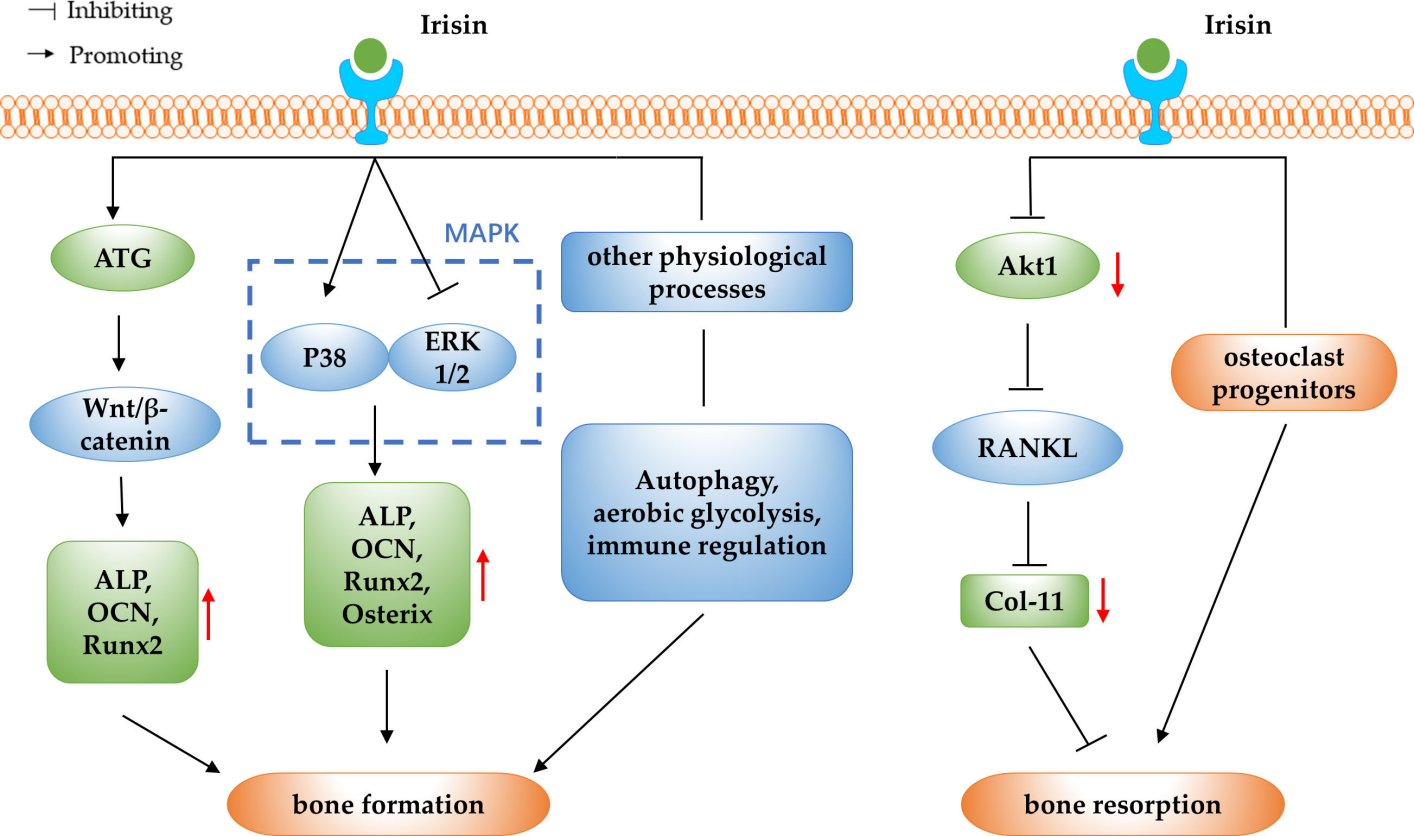
The schematic diagram of irisin regulating OP.

OP is a disorder of decreased bone mass, microarchitectural deterioration, and fragility fractures. With the acceleration of the ageing process, OP has become one of the chronic diseases that seriously endanger public health (66). In normal bone tissue, the bone formation and the bone resorption are in dynamic balance, and OP develops when bone resorption exceeds bone formation (67). In addition to genetic factors, hormones, nutritional status, exercise and lifestyle factors that have an impact on the development of OP, some myokines have been found to be associated with the development of OP in recent years (68). Irisin is a novel myokine and the correlation between serum irisin levels and BMD and OP has been clinically studied in different populations. One study reported that serum irisin levels were significantly decreased in postmenopausal women with OP and in middle-aged and elderly men with OP, and the lower the serum irisin level, the higher the risk of OP fragility fracture (69–71). A study of 43 elderly Chinese men with OP showed a positive correlation between irisin levels and BMD in geriatric Chinese men (72). The above study showed that serum irisin levels were decreased and positively correlated with BMD in OP patients, suggesting that irisin may be used as a predictor and prognostic biomarker for OP. Further studies have found that irisin inhibits the decrease in BMD and prevents bone loss (73–75). Kim et al. (18) reported in 2018 that irisin could interact with the αV/β5 integrin and thus promote bone remodeling, which had a positive effect on stopping bone loss. The above studies suggest that irisin may be a potential therapeutic target for bone loss and OP. Based on the previous findings, researchers have further explored the specific molecular mechanisms of irisin regulation of OP in terms of its regulatory effects on bone resorption and bone formation (as shown in **Figure 2**).

### 4.1 Irisin regulate OP by promoting bone formation

The osteogenic potential of irisin has been demonstrated by assessing its effect on yet-undifferentiated marrow stromal cells: r-irisin increased osteoblast differentiation in bone marrow stromal cells, as evidenced by an increase in the number of Alp-positive (Cfu-f) and von Kossa-positive (mineralized) colonies (Cfu-ob) (10). *In vivo* experimental studies have shown that, irisin deficient mice showed a lower bone density and significantly delayed bone development and mineralization from early‐stage to adulthood (28). In addition, irisin intraperitoneal (IP) administration resulted in increased trabecular and cortical bone thickness and osteoblasts numbers (76). These studies suggest that irisin can increase osteoblast differentiation and promote bone formation, and further studies are needed to elucidate the molecular mechanisms underlying the promotion of bone formation by irisin and to determine the potential therapeutic effects of irisin in OP.

#### 4.1.1 Irisin promote bone formation by activating the Wnt/β-catenin pathway

Wnt proteins are a large family of secreted glycoproteins that mediate the Wnt/β-catenin signalling pathway, which is essential in many life processes (77). Bone formation and reconstruction have been shown to be associated with the Wnt/β-catenin signalling pathway, and when Wnt/β-catenin signalling is disturbed, skeletal disease may occur (78). Sost is a Wnt signalling inhibitor that binds to the Wnt co-receptor low density lipoprotein receptor-related protein 5/6 (LRP5/6) and inhibits the activation of the Wnt/β-catenin pathway. It has been reported that irisin can promote bone formation in osteoblasts by inhibiting Sost expression, suggesting a potential role for irisin in promoting osteogenic differentiation by regulating the Wnt/β-catenin pathway (10, 38, 79). *In vitro* experiments have shown that irisin can activate the Wnt/β-catenin signalling pathway and promote osteogenic differentiation in BMSCs (28). In addition, Chen et al. (11) found in simulated microgravity experiments in mice that recombinant irisin could positively regulate osteoblast differentiation by increasing β-catenin expression in simulated microgravity, which suggested an activating effect of irisin on the Wnt/β-catenin signalling pathway in the promotion of osteogenesis. Another study reported that irisin may upregulate autophagy by increasing the Atg12-Atg5-Atg16L complex, and with the increasing level of autophagy, osteogenesis and the Wnt/β-catenin signal pathway were also enhanced, which ultimately promote osteogenic differentiation of bone marrow mesenchymal stem cells (80).

In summary, irisin is able to promote bone formation by targeting the Wnt/β-catenin pathway, finally leading to the treatment of OP. It is worth considering that Sost competitively inhibits the activation of Wnt/β-catenin signalling while competitively binding to type I and type II receptors to inhibit the bone morphogenetic protein (BMP) signalling pathway, thereby inhibiting osteoblast differentiation and bone formation (81). This could be an entry point to explore the interaction between Wnt and BMP signalling pathways in the promotion of osteogenic differentiation by irisin in the future.

#### 4.1.2 Irisin promote bone formation by modulating the MAPK pathway

The MAPK signalling pathway is one of the important signalling pathways for the directed proliferation and differentiation of BMSCs into osteoblasts, including two parallel and opposing signalling pathways, ERKl/2 and p38MAPK. P38 MAPK signalling pathway can promote osteoblast differentiation, while ERKl/2 signalling pathway inhibits osteoblast differentiation. The synergistic balance between the two pathways will determine whether BMSCs differentiate into osteoblasts or not (82–84). In a study using anti-FNDC5 antibody and irisin ELISA kit, it was found that irisin increased mRNA expression of osteogenic transcription factors including Runx2 and Osterix, as well as early markers of osteoblast differentiation, ALP and COL1α1, via activation of p38 MAPK and ERK. Furthermore, inhibition of the p38MAPK or ERK signalling pathways resulted in the elimination of the proliferation and up-regulatory effects of irisin, thus confirming the direct effects of irisin on osteoblasts via these pathways (85). *In vitro* experiments using an immortalized mouse cementoblast cell line OCCM-30 revealed that Runx2, osterix, ALP, and osteocalcin were obviously up-regulated under irisin stimulation as well as the activity of p38 MAPK pathway, and OCCM-30 cell proliferation was enhanced when treated with high-dose irisin for long time (86). This study confirmed that irisin can promote osteoblast differentiation via the p38MAPK pathway. In addition, Zhang et al. (87) investigated the effect of irisin on the osteogenic differentiation of rat BMSCs under static force and mechanical strain. The results showed that irisin can promote the proliferation and osteogenic differentiation of BMSCs under static force and mechanical strain, possibly via the P38 and ERK1/2 MAPK pathways.

In summary, MAPK pathway plays an important regulatory role in the pathogenesis of OP, and irisin can promote the proliferation and differentiation of osteoblasts and BMSCs by regulating p38 and ERK1/2MAPK signalling pathways, which may provide a new idea for the prevention and treatment of OP. Further studies are needed to explore the role of irisin on the synergistic relationship between the two signalling pathways, to reveal the specific regulatory processes involved, and to provide a theoretical basis for clinical exploration of the use of irisin to improve osteogenic effects.

#### 4.1.3 Irisin promote bone formation by involving in other physiological processes

Irisin regulates bone formation in relation to the mechanism of autophagy. Autophagy is an important mechanism commonly found in various eukaryotic life processes to remove excess or damaged biomolecules, through which organisms maintain intracellular metabolic homeostasis and internal environmental homeostasis, which is a process that plays an important role in the removal of intracellular waste, tissue remodeling and growth and development (88). In an *in vitro* study on osteogenic differentiation of BMSCs derived from aged mice, it was discovered that irisin increased autophagic activity. The study also evaluated the expression levels of Runx2, Osterix, ALP, and COL-I, and found that the upregulation of osteogenic transcription factors by irisin was significantly reduced after autophagy was inhibited. This suggests that irisin promotes osteogenic differentiation of BMSCs from senile mice by activating autophagy (89). In addition, aerobic glycolysis has been shown to promote bone mass gain *in vivo*, and irisin may also stimulate early osteoblast differentiation by activating aerobic glycolysis (90). A study by Zhang et al. (91) found that the promotion of r-irisin on the proliferation and differentiation of osteoblast lineage cells are preferentially through aerobic glycolysis, as indicated by the enhanced abundance of representative enzymes such as lactate dehydrogenase A (LDHA) and pyruvate dehydrogenase kinase 1 (PDK1), together with increased lactate levels. The results of this study suggest that r-irisin promotes osteoblast proliferation and differentiation by means of aerobic glycolysis. Other studies have shown that irisin can promote osteogenesis through immunomodulatory effects, it can promote osteoblast differentiation through immune effects generated by macrophage polarization. The direct co-cultured test of Raw264.7 macrophages and pre-osteoblastic MC3T3-E1 cells showed that irisin-treated M0 and M1 macrophages promoted osteo-genesis with obvious formation of mineralized particles, and this effect might be associated with activation of adenosine monophosphate protein kinase (AMPK) (92).

In conclusion, the effects of irisin on bone formation involve autophagy, aerobic glycolysis and immune regulation, reflecting the diversity of pathways through which irisin promotes osteogenesis. Further studies are needed to explore the other mechanisms involved in bone formation and to lay the foundation for its application in the prevention and treatment of OP.

### 4.2 Irisin regulate OP by modulating bone resorption

Current mainstream researches suggest that irisin may inhibit bone resorption in addition to its osteogenic effects, but there are also studies that suggest that irisin may promote bone resorption. *In vivo* experiments demonstrated that irisin deficiency resulted in increased expression of osteoclast-related genes, such as tartrate resistant acid phosphatase (TRAP), MMP9, and nuclear factor of activated T cells c1 (NFATc1), indicating the inhibitory effects and mechanisms of irisin on osteoclastogenesis (28). *In vitro* studies showed that irisin inhibited the receptor activator of nuclear factor-kB ligand (RANKL) induced Akt cascade response by down-regulating serine/threonine kinase 1 (Akt1) phosphorylation levels, which inhibited NFATc1 activation required for osteoclast differentiation and osteoclast formation (76). Furthermore, the study using 2 types of osteoclast precursor cells, RAW264.7 cells and mouse bone marrow monocytes, showed that irisin was able to promote the proliferation of both cells by activating the p38 and JNK signaling pathways, while inhibiting the RANKL-mediated NF-κB pathway to re-strict osteoclast differentiation (93). However, paradoxically, Estell et al. (94) reported through *in vitro* experiments that irisin acts directly on osteoclast progenitors to increase differentiation and promote bone resorption. The potential reason for this discrepant result could be that the animal experiments in this study were conducted with FNDC5 transgenic mice at different months of age, and there is temporal variability in the osteogenic and osteoclastic characteristics of mice at different developmental stages. In addition, the study design and the specific study subjects may also differ in their results, for example, the different strains of mice selected for the animal experiments and the different cell types applied in the cell experiments may have an impact on the results.

In summary, irisin inhibits the cellular differentiation of osteoclasts mainly by inhibiting the function of RANKL, but some researchers have proposed a different viewpoint, suggesting that irisin has a facilitative effect on bone resorption. As the number of relevant studies is small, no definite conclusion can be drawn for the time being, therefore the regulatory effect of irisin on bone resorption can be the focus of future studies.

## 5 Irisin and other bone diseases

### 5.1 Irisin and RA

RA is a chronic systemic autoimmune disease characterized by arthropathy, with pathological manifestations such as massive inflammatory cell infiltration in the synovial interstitium, destruction of cartilage and bone tissue, and persistent proliferation of synovial cells, with chronic inflammation and destruction of synovial joints being the main pathological changes in RA (95). One study showed that serum irisin levels are reduced in RA patients and the decrease of irisin production are related to disease activity, bone mineral density and skeletal muscle mass (96). This study suggests that the lower the level of irisin, the greater the rheumatoid activity and the more severe the bone loss and skeletal muscle mass loss. Therefore, irisin could be used as an indicator for the assessment of RA disease activity and concurrent OP and skeletal muscle loss. Other researchers have shown that irisin levels are lower in RA patients than in normal subjects, and irisin levels were decreased in RA patients with poor sleep quality compared to RA patients with good sleep quality and healthy controls. Irisin levels correlate with RA sleep quality, disease duration and disease activity, indicating a possible association of decreased serum irisin with sleep impairment in RA patients (97). Further studies have shown that irisin can modulate immuno-inflammatory in experimentally induced RA in rats by inhibiting the expression of receptor-interacting protein kinase 1 (RIPK1) and mixed-lineage kinase domain-like protein (MLKL) expression, thereby ameliorating the pathogenesis of RA (98). In addition, irisin reduced the expression of pro-inflammatory cytokines in tissues and the level of oxidative stress in the organism, suggesting that irisin could be involved in the progression of RA by regulating inflammation and oxidative stress.

In summary, serum irisin levels are decreased in RA patients, so irisin may be used as a predictor of RA. In addition, animal studies have demonstrated the therapeutic effect of irisin on RA, which is essentially due to its anti-inflammatory and antioxidant effects. Further *in vitro* experiments should be conducted to explore the interaction mechanisms between irisin and pro-inflammatory cytokines such as high mobility group protein B1 (HMGB1), monocyte chemotactic protein 1 (MCP1) and TNF-α. To support the use of irisin as a novel therapeutic agent to alleviate the development of RA.

### 5.2 Irisin and bone fracture

Bone fracture healing is a complex event that involves the coordination of different processes: initial inflammatory response, soft and hard callus formation, initial bony union and bone remodeling (99). Investigating the risk factors for bone fracture and the causes that influence bone fracture healing has been a keen subject of research. Researchers enrolled 160 older women (ages, 70-90 y) with minimal trauma hip fractures (MTHFs) and 160 age-matched women without fracture serving as controls, and the results of the test showed that the subjects in the MTHFs group had lower serum irisin levels compared to the control group, indicating that increased risk of hip fracture in older adults at low serum irisin levels, suggesting that irisin may be a useful indicator to help identify the risk of MTHFs in older women (100). In addition, Zhang et al. (101) proposed that the decrease of serum irisin levels in elderly patients with femoral shaft fracture after compression plate internal fixation is related to fracture nonunion, which can be used as a predictive index of fracture union evaluation. Other studies have reported that irisin levels in the blood increased in the bone fracture union process, and as irisin receptors were in human bone tissue, bone fracture union was affected (102). Recently, it has been shown that systemic administration of intermittent low doses of irisin accelerates bone fracture healing in mice by promoting bone formation and mineralization (103). Researchers administered r-irisin intraperitoneally to male rats immediately after bone fracture at 8 weeks of age and evaluated the effect of irisin treatment. The results showed that irisin induced a shift from cartilage to bone healing tissue by decreasing the expression of sex-determining region Y box protein 9 (SOX9) and increasing the expression of RUNX2, an important transcription factor that regulates osteoblast differentiation. The bone healing tissue area and bone volume were larger and the bone mineral content was higher in rat injected intraperitoneally with r-irisin compared to the control group. This experiment suggests that irisin can promote bone formation in bone scabs and accelerate the process of bone fracture repair, and it may be a novel pharmacological modulator of bone fracture healing. Similarly, Oranger et al. showed in a mouse model of tibial fracture that systemic administration of a low intermittent dose of irisin can accelerate the healing process by acting early in the post-fracture stage. This is due to the ability of this myokine to simultaneously switch off inflammation, activate angiogenesis, and initiate the degradation and replacement of cartilaginous matrix (104). In addition, in the study investigating the treatment of osteoporotic fractures in the ovariectomized rats with irisin combined with extracorporeal shock waves (ESW), it was found that both ESW and irisin alone could induce bone scab production and promote bone fracture healing, and the combined effect of them on bone fracture healing was more pronounced, perhaps because irisin combined with ESW might activate the nuclear factor-erythroid 2-related factor 2 (Nrf2)/heme oxygenase 1 (HO-1) pathway to promote the expression of vascular endothelial cell growth factor (VEGF) and BMP2 proteins, which induces angiogenesis, promotes blood circulation and accelerates bone scab formation, thus promotes fracture healing (105).

In summary, low serum levels of irisin increase the risk of bone fracture occurrence and bone fracture non-union, and *in vivo* administration of r-irisin promotes bone scab formation and accelerates fracture repair, so irisin may be used as a novel pharmaco-logical modulator to promote bone fracture healing, and in the future irisin may be used to eliminate non-union by applying it to non-union bones. In addition, synergistic effects of irisin with other therapeutic modalities are also of interest, which could open up new avenues for bone fracture healing.

### 5.3 Irisin and OS

OS is the most prevalent primary malignant tumor among human bone tumors, with typical clinical symptoms of pain, local masses, limitation of motion and pathological fractures, and it occurs in the long epiphyses of the extremities. It is extremely malignant, aggressive, rapidly growing and prone to early metastasis, and patients who develop early metastasis have an extremely poor healing process (106–108). Clinically, early amputation surgery supplemented by preoperative and postoperative chemotherapy is the main treatment option, but 80% of patients have metastatic lesions in their bodies at the time of diagnosis, resulting in poor outcomes, therefore, novel anti-cancer drugs and new treatment options need to be developed with OS cell growth inhibition as an entry point (109). It has been shown that irisin was significantly downregulated in the serums and tissues of osteosarcoma patients. In addition, irisin could inhibit the viability, migration, invasion, and EMT of osteosarcoma cells, and miR-214-3p could target FNDC5 to release its antitumor effects. Thus, irisin and miR-214-3p might become a new direction for the treatment of osteosarcoma patients in the future (110). The study by Kong et al. (111) found that irisin reversed the IL-6-induced epithelial-mesenchymal transition (EMT) in osteosarcoma cells by regulating the expression of E-cadherin, N-cadherin, vimentin, fibronectin, MMP-2, MMP-7 and MMP-9 through the signal transducers and activators of transcription 3 (STAT3)/Snail signaling pathway, implying that irisin may be a promising drug for OS treatment.

In summary, irisin treatment significantly suppressed the proliferation, migration and invasion of OS cells and has the potential to treat OS. However, the current research is focused on *in vitro* experiments, and large number of *in vivo* experiments are needed to verify and support in the future, to explore the specific molecular mechanism of OS regulation by irisin, and to extend the results to the clinic for systematic clinical studies to lay the foundation for the development of novel anti-tumor drugs.

## 6 Conclusion

Summarizing studies in recent years finds that the emerging myokine irisin is inextricably linked to bone health problems, and low serum irisin levels may increase the risk of fracture and lead to a series of bone diseases such as OP, RA and OS, so irisin may serve as a predictor of bone diseases. In addition, irisin can affect the physiological function of bone tissue cells through multiple signalling pathways and thus play a protective role in bone diseases. On the one hand, irisin can inhibit inflammatory responses by targeting MAPK and NF-κB signalling pathways, improve chondrocyte metabolism, enhance chondrocyte activity, and protect articular cartilage from inflammatory responses, thereby alleviating OA progression. On the other hand, irisin regulates Wnt/β-catenin and MAPK signalling pathways to induce proliferation and differentiation of osteoblasts and osteocytes, inhibit apoptosis of osteoblasts and osteocytes, maintain the physiological activity of osteocytes, and promote bone formation, meanwhile, it inhibits the function of RANKL, suppresses osteoclast differentiation, and slows down bone resorption, ultimately achieving the purpose of treating OP. However, some researchers have proposed a different perspective, suggesting that irisin may play a facilitating role in bone resorption. Further research is required to explain the reason for this controversy. In addition to the targeted regulation of the above-mentioned signalling pathways, irisin can also attenuating the development of OA and OP by protecting mitochondrial function, regulating autophagy, aerobic glycolysis and immune regulation and other physiological processes. In addition, irisin also plays a role in other bone diseases, for example, irisin can promote bone fracture healing, improve the pathogenesis of RA and OS, and has the potential to treat RA and OS, and the possibility of irisin treatment for different types of bone diseases can be further investigated in the future (as shown in **Table 1**).

**Table 1:** Irisin regulate bone disease.

Authors	Bone Disease	Pathway	Target cells
He et al. (39)	OA	ERK	Osteocytes
Feng et al. (41)	OA	P38 MAPK	Chondrocytes
Vadalàet al. (44)	OA	JNK	Chondrocytes
Jia et al. (53)	OA	NF-κB	Chondrocytes
Wang et al. (65)	OA	Mitochondrial	Chondrocytes
Zhu et al. (28)	OP	Wnt/β-catenin	BMSCs
Chen et al. (11)	OP	Wnt/β-catenin	Osteoblast
Chen et al. (80)	OP	Wnt/β-catenin	BMSCs
Zhang et al. (87)	OP	P38 MAPK, ERK1/2	BMSCs
Lei et al. (89)	OP	Autophagy	BMSCs
Zhang et al. (91)	OP	Aerobic glycolysis	Osteoblast
Ye et al. (92)	OP	Immune regulation	Osteoblast
Zhang et al. (76)	OP	NF-κB	Osteoclast
Ma et al. (93)	OP	NF-κB	Osteoclast
Estell et al. (94)	OP	/	Osteoclast
Raafat et al. (98)	RA	RIPK1, MLKL	Inflammatory cell
Colucci et al. (103)	Bone fracture	SOX9, Runx2	/
Oranger et al. (104)	Bone fracture	TNFα, MIP-1α, MMP-13, BMP2	/
Heqiu et al. (105)	Bone fracture	Nrf2/HO-1	/

/, no content; OA, osteoarthritis; OP, osteoporosis; RA, rheumatoid arthritis; ERK, extracellular regulated kinase; p38MAPK, p38 mitogen-activated protein kinase; JNK, c-Jun N-terminal kinase; NF-κB, nuclear factor kappa-light-chain-enhancer of activated B cells; RIPK1, receptor-interacting protein kinase 1; MLKL, mixed-lineage kinase domain-like protein; SOX9, sex-determining region Y box protein 9; Runx2, Runt-related Transcription Factor 2; TNFα, tumor necrosis factor α; MIP-1α, macrophage inflammatory protein 1α; MMP-13, matrix metalloproteinase-13; BMP2, bone morphogenetic protein 2; Nrf2/HO-1, nuclear factor-erythroid 2-related factor 2/heme oxygenase 1; BMSCs, bone marrow mesenchymal stem cells.

However, the relationship between irisin and bone diseases still has several urgent problems to be solved: first, studies have confirmed that irisin can affect the physiological function of bone tissue cells through multiple signaling pathways, but only the αV/β5 integrin has been identified as the receptor for irisin on bone cells, and other target receptors are not yet known. Secondly, most of the current studies focus on animal experiments and *in vitro* experiments, lacking the validation and support of clinical data. In the future, large number of studies are needed to explore the specific therapeutic effects and adverse effects of irisin on bone diseases, providing a solid theoretical basis for its safe and effective application in clinical work. Finally, the investigation of the relationship between irisin and bone health is an emerging field in recent years, although some signaling pathways related to the physiological effects of irisin have been identified, but the specific mechanisms of these signaling pathways and action targets are not exhaustive and perfect, and even many aspects still have research gaps, and further in-depth studies are still needed in the future.

## Author contributions

Writing—original draft preparation, RZ and YC. Writing—review and editing, DW, CZ and HS. Funding acquisition, GN. All authors contributed to the article and approved the submitted version.

## References

[1] Rodan GA Martin TJ. Therapeutic approaches to bone diseases. Sci (New York N.Y (2000) ) 289:1508. doi: 10.1126/science.289.5484.1508 10968781

[2] Chotiyarnwong P McCloskey EV. Pathogenesis of glucocorticoid-induced osteoporosis and options for treatment. Nat Rev Endocrinol (2020) 16:437. doi: 10.1038/s41574-020-0341-0 32286516

[3] Brotto M Johnson ML. Endocrine crosstalk between muscle and bone. Curr osteoporosis Rep (2014) 12:135. doi: 10.1007/s11914-014-0209-0 PMC4374433 24667990

[4] Pedersen BK Febbraio MA. Muscles, exercise and obesity: skeletal muscle as a secretory organ. Nature reviews. Endocrinology (2012) 8:457. doi: 10.1038/nrendo.2012.49 22473333

[5] Kaji H. Effects of myokines on bone. BoneKEy Rep (2160) 5:826. doi: 10.1038/bonekey.2016.48 PMC4954587 27579164

[6] Hamrick MW. The skeletal muscle secretome: an emerging player in muscle-bone crosstalk. BoneKEy Rep (2012) 1:60. doi: 10.1038/bonekey.2012.60 23951457 PMC3727847

[7] Kirk B Feehan J Lombardi G Duque G. Muscle, bone, and fat crosstalk: the biological role of myokines, osteokines, and adipokines. Curr osteoporosis Rep (2020) 18:388. doi: 10.1007/s11914-020-00599-y 32529456

[8] Boström P Wu J Jedrychowski MP Korde A Ye L Lo JC. A PGC1-α-dependent myokine that drives brown-fat-like development of white fat and thermogenesis. Nature. (2012) 481:463. doi: 10.1038/nature10777 22237023 PMC3522098

[9] Colaianni G Sanesi L Storlino G Brunetti G Colucci S Grano M. Irisin and bone: from preclinical studies to the evaluation of its circulating levels in different populations of human subjects. Cells (2019) 8:451. doi: 10.3390/cells8050451 31091695 PMC6562988

[10] Colaianni G Cuscito C Mongelli T Pignataro P Buccoliero C Liu P. The myokine irisin increases cortical bone mass. Proc Natl Acad Sci USA (2015) 112:12157. doi: 10.1073/pnas.1516622112 26374841 PMC4593131

[11] Chen Z Zhang Y Zhao F Yin C Yang C Wang X. Recombinant irisin prevents the reduction of osteoblast differentiation induced by stimulated microgravity through increasing β-catenin expression. Int J Mol Sci (2020) 21:1259. doi: 10.3390/ijms21041259 32070052 PMC7072919

[12] Silvia C Graziana C Giacomina B Francesca F Gabriele M Giorgio M. Irisin prevents microgravity-induced impairment of osteoblast differentiation in *vitro* during the space flight CRS-14 mission. FASEB J (2020) 34:10096. doi: 10.1096/fj.202000216R 32539174

[13] Colaianni G Mongelli T Cuscito C Pignataro P Lippo L Spiro G. Irisin prevents and restores bone loss and muscle atrophy in hind-limb suspended mice. Sci Rep (2015) 7:2811. doi: 10.1038/s41598-017-02557-8 PMC5460172 28588307

[14] Roca-Rivada A Castelao C Senin LL Landrove MO Baltar J Belén Crujeiras A. FNDC5/irisin is not only a myokine but also an adipokine. PloS One (2013) 8:e60563. doi: 10.1371/journal.pone.0060563 23593248 PMC3623960

[15] Lee P Linderman JD Smith S Brychta RJ Wang J Idelson C. Irisin and FGF21 are cold-induced endocrine activators of brown fat function in humans. Cell Metab (2014) 19:302. doi: 10.1016/j.cmet.2013.12.017 24506871 PMC7647184

[16] Vaughan RA Garcia-Smith R Bisoffi M Conn CA Trujillo KA. Conjugated linoleic acid or omega 3 fatty acids increase mitochondrial biosynthesis and metabolism in skeletal muscle cells. Lipids Health disease. (2012) 11:142. doi: 10.1186/1476-511x-11-142 PMC3515476 23107305

[17] Aydin S Aydin S Kuloglu T Yilmaz M Kalayci M Sahin I. Alterations of irisin concentrations in saliva and serum of obese and normal-weight subjects, before and after 45 min of a Turkish bath or running. Peptides. (2014) 50:13. doi: 10.1016/j.peptides.2013.09.011 24096106

[18] Kim H Wrann CD Jedrychowski M Vidoni S Kitase Y Nagano K. Irisin mediates effects on bone and fat via αV integrin receptors. Cell. (2018) 175:1756. doi: 10.1016/j.cell.2018.10.025 30550785 PMC6298040

[19] Hynes RO. Integrins: bidirectional, allosteric signaling machines. Cell. (2002) 110:673. doi: 10.1016/s0092-8674(02)00971-6 12297042

[20] Memmo LM McKeown-Longo P. The alphavbeta5 integrin functions as an endocytic receptor for vitronectin. J Cell science. (1998) 111(Pt 4):425. doi: 10.1242/jcs.111.4.425 9443892

[21] Martinez Munoz IY Camarillo Romero EDS Garduno Garcia JJ. Irisin a novel metabolic biomarker: present knowledge and future directions. Int J endocrinology. (2018) 1:8. doi: 10.1155/2018/7816806 PMC6198573 30402097

[22] Liu J Hu Y Zhang H Xu Y Wang G. Exenatide treatment increases serum irisin levels in patients with obesity and newly diagnosed type 2 diabetes. J Diabetes its complications. (2016) 30:1555. doi: 10.1016/j.jdiacomp.2016.07.020 27503404

[23] Buscemi S Corleo D Buscemi C Giordano C. Does iris(in) bring bad news or good news? Eating weight disorders: EWD. (2018) 23:431. doi: 10.1007/s40519-017-0431-8 28933009

[24] Du XL Jiang WX Lv ZT. Lower circulating irisin level in patients with diabetes mellitus: A systematic review and meta-analysis. Hormone Metab Res = Hormon- und Stoffwechselforschung = Hormones metabolisme. (2016) 48:644. doi: 10.1055/s-0042-108730 27300472

[25] Deng X Huang W Peng J Zhu TT Sun XL Zhou XY. Irisin alleviates advanced glycation end products-induced inflammation and endothelial dysfunction via inhibiting ROS-NLRP3 inflammasome signaling. Inflammation. (2018) 41:260. doi: 10.1007/s10753-017-0685-3 29098483

[26] Peng J Deng X Huang W Yu JH Wang JX Wang JP. Irisin protects against neuronal injury induced by oxygen-glucose deprivation in part depends on the inhibition of ROS-NLRP3 inflammatory signaling pathway. Mol Immunol (2017) 91:185. doi: 10.1016/j.molimm.2017.09.014 28961497

[27] Zhang Y Mu Q Zhou Z Song H Zhang Y Wu F. Protective effect of irisin on atherosclerosis via suppressing oxidized low density lipo-protein induced vascular inflammation and endothelial dysfunction. PloS One (2016) 11:e0158038. doi: 10.1371/journal.pone.0158038 27355581 PMC4927070

[28] Zhu X Li X Wang X Chen T Tao F Liu C. Irisin deficiency disturbs bone metabolism. J Cell Physiol (2021) 236:664. doi: 10.1002/jcp.29894 32572964 PMC7722136

[29] Zhou X Cao H Yuan Y Wu W. Biochemical signals mediate the crosstalk between cartilage and bone in osteoar-thritis. BioMed Res Int (2020) 1:8. doi: 10.1155/2020/5720360 PMC7165323 32337258

[30] Goldring MB Berenbaum F. Emerging targets in osteoarthritis therapy. Curr Opin Pharmacol (2015) 22:51. doi: 10.1016/j.coph.2015.03.004 25863583 PMC4470796

[31] Mao Y Xu W Xie Z Dong Q. Association of irisin and CRP levels with the radiographic severity of knee osteoar-thritis. Genet testing Mol biomarkers. (2015) 20:86. doi: 10.1089/gtmb.2015.0170 26625129

[32] Li X Zhu X Wu H Van Dyke TE Xu X Morgan EF. Roles and mechanisms of irisin in attenuating pathological features of osteoarthritis. Front Cell Dev Biol (2021) 9:703670. doi: 10.3389/fcell.2021.703670 34650969 PMC8509718

[33] Posa F Zerlotin R Ariano A Cosola MD Colaianni G Fazio AD. Irisin role in chondrocyte 3D culture differentiation and its possible applications. Pharmaceutics. (2023) 15(2):585. doi: 10.3390/pharmaceutics15020585 36839906 PMC9961836

[34] Joshi S Platanias LC. Mnk kinase pathway: Cellular functions and biological outcomes. World J Biol Chem (2014) 5:321. doi: 10.4331/wjbc.v5.i3.321 25225600 PMC4160526

[35] Kimura H Yukitake H Suzuki H Tajima Y Gomaibashi K Morimoto S. The chondroprotective agent ITZ-1 inhibits interleukin-1beta-induced matrix metalloproteinase-13 production and suppresses nitric oxide-induced chondrocyte death. J Pharmacol Sci (2009) 110:201. doi: 10.1254/jphs.09076fp 19542681

[36] Lawrence RC Felson DT Helmick CG Arnold LM Choi H Deyo RA. Estimates of the prevalence of arthritis and other rheumatic conditions in the United States. Part II. Arthritis rheumatism. (2008) 58:26. doi: 10.1002/art.23176 18163497 PMC3266664

[37] Boileau C Martel-Pelletier J Brunet J Schrier D Flory C Boily M. PD-0200347, an alpha2delta ligand of the voltage gated calcium channel, inhibits in *vivo* activation of the Erk1/2 pathway in osteoarthritic chondrocytes: a PKCalpha dependent effect. Ann rheumatic diseases. (2006) 65:573. doi: 10.1136/ard.2005.041855 PMC1798126 16249226

[38] Storlino G Colaianni G Sanesi L Lippo L Brunetti G Errede M. Irisin prevents disuse-induced osteocyte apoptosis. J Bone mineral Res Off J Am Soc Bone Mineral Res (2020) 35:766. doi: 10.1002/jbmr.3944 31826311

[39] He Z Li H Han X Zhou F Du J Yang Y. Irisin inhibits osteocyte apoptosis by activating the Erk signaling pathway in *vitro* and attenuates ALCT-induced osteoarthritis in mice. Bone. (2020) 141:115573. doi: 10.1016/j.bone.2020.115573 32768686

[40] Kapoor M Martel-Pelletier J Lajeunesse D Pelletier JP Fahmi H. Role of proinflammatory cytokines in the patho-physiology of osteoarthritis. Nat Rev Rheumatol (2010) 7:33. doi: 10.1038/nrrheum.2010.196 21119608

[41] Feng Z Xiaojuan S Zifan Z Linlin F Yonfeng Z. Experimental study on the regulation of p38MAPK pathway by irisin-targeted TDP-43 for the relief of osteoarthritis. Anatomical Stud (2021) 43:584. doi: 10.3969/j.issn.1671-0770

[42] Yoon HS Kim HA. Prologation of c-Jun N-terminal kinase is associated with cell death induced by tumor necrosis factor alpha in human chondrocytes. J Korean Med science. (2004) 19:567. doi: 10.3346/jkms.2004.19.4.567 PMC2816892 15308849

[43] Mazur-Bialy AI Pocheć E Zarawski M. Anti-inflammatory properties of irisin, mediator of physical activity, are connected with TLR4/myD88 signaling pathway activation. Int J Mol Sci (2017) 18:701. doi: 10.3390/ijms18040701 28346354 PMC5412287

[44] Vadalà G Di Giacomo G Ambrosio L Cannata F Cicione C Papalia R. Irisin recovers osteoarthritic chondrocytes *in vitro* . Cells. (2020) 9:1478. doi: 10.3390/cells9061478 32560375 PMC7348865

[45] Jimi E Fei H Nakatomi C. NF-κB signaling regulates physiological and pathological chondrogenesis. Int J Mol Sci (2019) 20:6275. doi: 10.3390/ijms20246275 31842396 PMC6941088

[46] Ji B Guo W Ma H Xu B Mu W Zhang Z. Isoliquiritigenin suppresses IL-1β induced apoptosis and inflammation in chondrocyte-like ATDC5 cells by inhibiting NF-κB and exerts chondroprotective effects on a mouse model of anterior cruciate ligament transection. Int J Mol Med (2017) 40:1709. doi: 10.3892/ijmm.2017.3177 29039445 PMC5716454

[47] Ostojic M Zevrnja A Vukojevic K Soljic V. Immunofluorescence Analysis of NF-kB and iNOS Expression in Different Cell Populations during Early and Advanced Knee Osteoarthritis. Int J Mol Sci (2021) 22:6461. doi: 10.3390/ijms22126461 34208719 PMC8233870

[48] Zhang Z Zhang C Zhang S. Irisin activates M1 macrophage and suppresses Th2-type immune response in rats with pelvic inflammatory disease. Evidence-Based complementary Altern Med eCAM. (2022) 1:7. doi: 10.1155/2022/5215915 PMC8853798 35186099

[49] Li Q Tan Y Chen S Xiao X Zhang M Wu Q. Irisin alleviates LPS-induced liver injury and inflammation through inhibition of NLRP3 inflammasome and NF-κB signaling. J receptor Signal transduction Res (2020) 41:294. doi: 10.1080/10799893.2020.1808675 32814473

[50] Jin YH Li ZY Jiang XQ Wu F Li ZT Chen H. Irisin alleviates renal injury caused by sepsis via the NF-κB signaling pathway. Eur Rev Med Pharmacol Sci (2020) 24:6470. doi: 10.26355/eurrev_202006_21546 32572945

[51] Huang Y Chen Q Jiang Q Zhao Z Fang J Chen L. Irisin lowers blood pressure in Zucker diabetic rats by regulating the functions of renal angiotensin II type 1 receptor via the inhibition of the NF-κB signaling pathway. Peptides. (2022) 147:170688. doi: 10.1016/j.peptides.2021.170688 34800756

[52] Li X Liu Y Liu Q Wang S Ma Y Jin Q. Recombinant human irisin regulated collagen II, matrix metalloproteinase-13 and the Wnt/β-catenin and NF-κB signaling pathways in interleukin-1β-induced human SW1353 cells. Exp Ther Med (2020) 19:2879. doi: 10.3892/etm.2020.8562 32256772 PMC7086223

[53] Jia S Yang Y Bai Y Wei Y Zhang H Tian Y. Mechanical stimulation protects against chondrocyte pyroptosis through irisin-induced suppression of PI3K/akt/NF-κB signal pathway in osteoarthritis. Front Cell Dev Biol (2022) 10:797855. doi: 10.3389/fcell.2022.797855 35356271 PMC8959944

[54] Hussain S Sun M Min Z Guo Y Xu J Mushtaq N. Down-regulated in OA cartilage, SFMBT2 contributes to NF-κB-mediated ECM degradation. J Cell Mol Med (2018) 22:5753. doi: 10.1111/jcmm.13826 30133133 PMC6201222

[55] Lin C Shao Y Zeng C Zhao C Fang H Wang L. Blocking PI3K/AKT signaling inhibits bone sclerosis in subchondral bone and attenuates post-traumatic osteoarthritis. J Cell Physiol (2018) 233:6135. doi: 10.1002/jcp.26460 29323710

[56] Sueishi T Akasaki Y Goto N Kurakazu I Toya M Kuwahara M. GRK5 inhibition attenuates cartilage degradation via decreased NF-κB signaling. Arthritis Rheumatol (Hoboken N.J.). (2020) 72:620. doi: 10.1002/art.41152 31696655

[57] Cillero-Pastor B Rego-Pérez I Oreiro N Fernandez-Lopez C Blanco FJ. Mitochondrial respiratory chain dysfunction modulates metalloproteases -1, -3 and -13 in human normal chondrocytes in culture. BMC musculoskeletal Disord (2013) 14:235. doi: 10.1186/1471-2474-14-235 PMC3750811 23937653

[58] Coleman MC Goetz JE Brouillette MJ Seol D Willey MC Petersen EB. Targeting mitochondrial responses to intra-articular fracture to prevent posttraumatic osteoarthritis. Sci Trans Med (2018) 10:eaan5372. doi: 10.1126/scitranslmed.aan5372 PMC5987523 29437147

[59] Blanco FJ Fernández-Moreno M. Mitochondrial biogenesis: a potential therapeutic target for osteoarthritis. Osteoarthritis cartilage. (2020) 28:1003. doi: 10.1016/j.joca.2020.03.018 32417558

[60] Fernández-Moreno M Soto-Hermida A Vázquez-Mosquera ME Cortés-Pereira E Relaño S Hermida-Gómez T. Mitochondrial DNA haplogroups in-fluence the risk of incident knee osteoarthritis in OAI and CHECK cohorts. A meta-analysis and functional study. Ann rheumatic diseases. (2016) 76:1114. doi: 10.1136/annrheumdis-2016-210131 27919866

[61] López de FP Lotz MK Blanco FJ Caramés B. Autophagy activation and protection from mitochondrial dysfunction in human chondrocytes. Arthritis Rheumatol (2015) 67(4):966–76. doi: 10.1002/art.39025 PMC4380780 25605458

[62] He W Wang P Chen Q Li C. Exercise enhances mitochondrial fission and mitophagy to improve myopathy following critical limb ischemia in elderly mice via the PGC1a/FNDC5/irisin pathway. Skeletal muscle. (2020) 10:25. doi: 10.1186/s13395-020-00245-2 32933582 PMC7490877

[63] Bi J Zhang J Ren Y Du Z Li Q Wang Y. Irisin alleviates liver is-chemia-reperfusion injury by inhibiting excessive mitochondrial fission, promoting mitochondrial biogenesis and decreasing oxidative stress. Redox Biol (2019) 20:296. doi: 10.1016/j.redox.2018.10.019 30388684 PMC6216086

[64] Jiang X Cai S Jin Y Wu F He J Wu X. Irisin attenuates oxidative stress, mitochondrial dysfunction, and apoptosis in the H9C2 cellular model of septic cardiomyopathy through augmenting fundc1-dependent mitophagy. Oxid Med Cell longevity. (2019) 1:9. doi: 10.1155/2021/2989974 PMC8390168 34457111

[65] Wang FS Kuo CW Ko JY Chen YS Wang SY Ke HJ. Irisin mitigates oxidative stress, chondrocyte dysfunction and osteoarthritis development through regulating mitochondrial integrity and autophagy. Antioxidants (Basel Switzerland). (2020) 9:810. doi: 10.3390/antiox9090810 32882839 PMC7555738

[66] Lane JM Russell L Khan SN. Osteoporosis. Clin orthopaedics related Res (2000) 372:139. doi: 10.1097/00003086-200003000-00016 10738423

[67] Ikeda K. Osteocytes in the pathogenesis of osteoporosis. Geriatrics gerontology Int (2008) 8:213. doi: 10.1111/j.1447-0594.2008.00481.x 19149831

[68] Li G Zhang L Wang D LA. I Jiang JX Xu H. Muscle-bone crosstalk and potential therapies for sar-co-osteoporosis. J Cell Biochem (2019) 120:14262. doi: 10.1002/jcb.28946 31106446 PMC7331460

[69] Badr Roomi A Nori W Mokram Hamed R. Lower serum irisin levels are associated with increased osteoporosis and oxidative stress in postmenopausal. Rep Biochem Mol Biol (2021) 10:13. doi: 10.52547/rbmb.10.1.13 34277864 PMC8279710

[70] Engin-Üstün Y Çağlayan EK Göçmen AY Polat MF. Postmenopausal osteoporosis is associated with serum chemerin and irisin but not with apolipoprotein M levels. J menopausal Med (2016) 22:76. doi: 10.6118/jmm.2016.22.2.76 27617241 PMC5016507

[71] Zhou K Qiao X Cai Y Li A Shan D. Lower circulating irisin in middle-aged and older adults with osteoporosis: a systematic review and meta-analysis. Menopause (New York N.Y.). (2019) 26:1302. doi: 10.1097/gme.0000000000001388 31688577

[72] Zhang J Huang X Yu R Wang Y Gao C. Circulating irisin is linked to bone mineral density in geriatric Chinese men. Open Med (Warsaw Poland). (2020) 15:763. doi: 10.1515/med-2020-0215 PMC7706135 33313413

[73] Iemura S Kawao N Okumoto K Akagi M Kaji H. Role of irisin in androgen-deficient muscle wasting and osteopenia in mice. J Bone mineral Metab (2020) 38:161. doi: 10.1007/s00774-019-01043-7 31494773

[74] Morgan EN Alsharidah AS Mousa AM Edrees HM. Irisin has a protective role against osteoporosis in ovari-ectomized rats. BioMed Res Int (2021) 1:10. doi: 10.1155/2021/5570229 PMC8096550 33997010

[75] Behera J Ison J Voor MJ Tyagi N. Exercise-linked skeletal irisin ameliorates diabetes-associated osteoporosis by inhibiting the oxidative damage-dependent miR-150-FNDC5/pyroptosis axis. Diabetes. (2022) 71:2777. doi: 10.2337/db21-0573 35802043 PMC9750954

[76] Zhang J Valverde P Zhu X Murray D Wu Y Yu L. Exercise-induced irisin in bone and systemic irisin administration reveal new regulatory mechanisms of bone metabolism. Bone Res (2016) 5:16056. doi: 10.1038/boneres.2016.56 PMC5605767 28944087

[77] Logan CY Nusse R. The Wnt signaling pathway in development and disease. Annu Rev Cell Dev Biol (2004) 20:781. doi: 10.1146/annurev.cellbio.20.010403.113126 15473860

[78] Glass DA 2nd Karsenty G. *In vivo* analysis of Wnt signaling in bone. Endocrinology. (2007) 148:2630. doi: 10.1210/en.2006-1372 17395705

[79] Mengting T Jing L Xueming Z Wenjing Z Xiangzhen H Huiyu H. Effect of irisin on Sost gene expression and os-teogenic differentiation of rat bone marrow mesenchymal stem cells. Chin J Oral Med Res (2019) 13:144. doi: 10.3877/cma.j.issn.1674⁃1366.2019.03.003

[80] Chen X Sun K Zhao S Geng T Fan X Sun S. Irisin promotes osteogenic differentiation of bone marrow mesenchymal stem cells by activating autophagy via the Wnt//β-catenin signal pathway. Cytokine. (2020) 136:155292. doi: 10.1016/j.cyto.2020.155292 32950809

[81] Spatz JM Wein MN Gooi JH Qu Y Garr JL Liu S. The wnt inhibitor sclerostin is up-regulated by mechanical unloading in os-teocytes in vitro. J Biol Chem (2015) 290:16744. doi: 10.1074/jbc.M114.628313 25953900 PMC4505423

[82] Lee HW Suh JH Kim HN Kim AY Park SY Shin CS. Berberine promotes osteoblast differentiation by Runx2 activation with p38 MAPK. J Bone mineral Res Off J Am Soc Bone Mineral Res (2008) 23:1227. doi: 10.1359/jbmr.080325 18410224

[83] Choi SC Kim SJ Choi JH Park CY Shim WJ Lim DS. Fibroblast growth factor-2 and -4 promote the prolif-eration of bone marrow mesenchymal stem cells by the activation of the PI3K-Akt and ERK1/2 signaling pathways. Stem Cells Dev (2008) 17:725. doi: 10.1089/scd.2007.0230 18788932

[84] Liao XB Zhou XM Li JM Yang JF Tan ZP Hu ZW. Taurine inhibits osteoblastic differentiation of vascular smooth muscle cells via the ERK pathway. Amino Acids (2008) 34:525. doi: 10.1007/s00726-007-0003-8 18060526

[85] Qiao X Nie Y Ma Y Chen Y Cheng R Yin W. Irisin promotes osteoblast proliferation and differentiation via activating the MAP kinase signaling pathways. Sci Rep (2016) 6:18732. doi: 10.1038/srep18732 26738434 PMC4704023

[86] Zhu J Wang Y Cao Z Du M Hao Y Pan J. Irisin promotes cementoblast differentiation via p38 MAPK pathway. Oral diseases. (2020) 26:974. doi: 10.1111/odi.13307 32068933

[87] Zewei Z Wenyang L Jun X Lihua Q. Study of osteogenic differentiation of BMSCs under the effect of static and tensile forces by irisin. J Chongqing Med University. (2019) 44:1134. doi: 10.13406/j.cnki.cyxb.002006

[88] Rubinsztein DC Mariño G Kroemer G. Autophagy and aging. Cell. (2011) 146:682. doi: 10.1016/j.cell.2011.07.030 21884931

[89] Lei S Keming D Yu Z Gang Z Cui L Yan L. *In vitro* studies on the promotion of osteotropic differentiation of aged mouse BMSCs by activation of autophagy by irisin. J Oral Maxillofac Prosthetics. (2020) 21:257. doi: 10.19748/j.cn.kqxf.1009-3761.2020.05.001

[90] Regan JN Lim J Shi Y Joeng KS Arbeit JM Shohet RV. Up-regulation of glycolytic metabolism is re-quired for HIF1α-driven bone formation. Proc Natl Acad Sci United States America. (2014) 111:8673. doi: 10.1073/pnas.1324290111 PMC4060724 24912186

[91] Zhang D Bae C Lee J Lee J Jin Z Kang M. The bone anabolic effects of irisin are through preferential stimulation of aerobic glycolysis. Bone. (2018) 114:150. doi: 10.1016/j.bone.2018.05.013 29775761

[92] Ye W Wang J Lin D Ding Z. The immunomodulatory role of irisin on osteogenesis via AMPK-mediated macrophage polarization. Int J Biol macromolecules. (2020) 146:25. doi: 10.1016/j.ijbiomac.2019.12.028 31843619

[93] Ma Y Qiao X Zeng R Cheng R Zhang J Luo Y. Irisin promotes proliferation but inhibits differentiation in osteoclast precursor cells. FASEB J: Off Publ Feder-ation Am Societies Exp Biol (2018) 17:fj201700983RR. doi: 10.1096/fj.201700983RR 29771602

[94] Estell EG Le PT Vegting Y Kim H Wrann C Bouxsein ML. Irisin directly stimulates osteoclastogenesis and bone resorption in *vitro* and in *vivo* . eLife. (2020) 9:e58172. doi: 10.7554/eLife.58172 32780016 PMC7444909

[95] Koenders MI van den Berg WB. Novel therapeutic targets in rheumatoid arthritis. Trends Pharmacol Sci (2015) 36:189. doi: 10.1016/j.tips.2015.02.001 25732812

[96] Shan W Shoulin Y Shengting R Xuwen C Guangyun X. Correlation of serum activin A and irisin levels with disease activity, bone mineral density and skeletal muscle mass in patients with rheumatoid arthritis. J Doubtful Diseases. (2022) 21:50. doi: 10.3969/j.issn.1671-6450.2022.01.010

[97] Gamal RM Mohamed ME Hammam N El Fetoh NA Rashed AM Furst DE. Preliminary study of the association of serum irisin levels with poor sleep quality in rheumatoid arthritis patients. Sleep Med (2020) 67:71. doi: 10.1016/j.sleep.2019.10.021 31918120

[98] Raafat Ibrahim R Shafik NM El-Esawy RO El-Sakaa MH Arakeeb HM El-Sharaby RM. The emerging role of irisin in experimentally induced arthritis: a recent update involving HMGB1/MCP1/Chitotriosidase I-mediated necroptosis. Redox Rep Commun Free Radical Res (2022) 27:21. doi: 10.1080/13510002.2022.2031516 PMC8803109 35094663

[99] Giganti MG Tresoldi I Masuelli L Modesti A Grosso G Liuni FM. Fracture healing: from basic science to role of nutrition. Front bioscience (Landmark edition). (2014) 19:1162. doi: 10.2741/4273 24896342

[100] Yan J Liu HJ Guo WC Yang J. Low serum concentrations of Irisin are associated with increased risk of hip fracture in Chinese older women. Joint Bone spine. (2018) 85:353. doi: 10.1016/j.jbspin.2017.03.011 28408276

[101] Yu Z Guolin M Honghong W. Relationship between serum irisin and hydrogen sulfide levels and fracture healing after internal fixation with compression plates in elderly patients with femoral stem fractures. Chin Med (2022) 17:1221. doi: 10.3760/j.issn.1673-4777.2022.08.023

[102] Serbest S Tiftikçi U Tosun HB Kısa Ü. The irisin hormone profile and expression in human bone tissue in the bone healing process in patients. Med Sci monitor Int Med J Exp Clin Res (2017) 23:4278. doi: 10.12659/msm.906293 PMC5597035 28869754

[103] Colucci SC Buccoliero C Sanesi L Errede M Colaianni G Annese T. Systemic administration of recombinant irisin accelerates fracture healing in mice. Int J Mol Sci (2021) 22:10863. doi: 10.3390/ijms221910863 34639200 PMC8509717

[104] Oranger A Zerlotin R Buccoliero C Sanesi L Storlino G Schipani E. Irisin modulates inflammatory, angiogenic, and osteogenic factors during fracture healing. Int J Mol Sci (2023) 24(3):1809. doi: 10.3390/ijms24031809 36768133 PMC9915346

[105] Heqiu X Li L Zifang Y. Effect of Iris and in *vitro* shock waves on fracture healing in devitalized rats. Chin J Orthopaedic Surgery. (2022) 30:732. doi: 10.3977/j.issn.1005-8478.2022.08.12

[106] Roessner A Lohmann C Jechorek D. Translational cell biology of highly malignant osteosarcoma. Pathol Int (2021) 71:291. doi: 10.1111/pin.13080 33631032

[107] Mirabello L Troisi RJ Savage SA. Osteosarcoma incidence and survival rates from 1973 to 2004: data from the Sur-veillance, Epidemiology, and End Results Program. Cancer. (2009) 115:1531. doi: 10.1002/cncr.24121 19197972 PMC2813207

[108] Rickel K Fang F Tao J. Molecular genetics of osteosarcoma. Bone. (2017) 102:69. doi: 10.1016/j.bone.2016.10.017 27760307 PMC5393957

[109] Moore DD Luu HH. Osteosarcoma. Cancer Treat Res (2014) 162:65. doi: 10.1007/978-3-319-07323-1_4 25070231

[110] Cheng G Xu D Chu K Cao Z Sun X Yang Y. The effects of miR-214-3p and Irisin/FNDC5 on the biological behavior of osteosarcoma cells. Cancer biotherapy radiopharmaceuticals. (2020) 35:92. doi: 10.1089/cbr.2019.2933 32073886

[111] Kong G Jiang Y Sun X Cao Z Zhang G Zhao Z. Irisin reverses the IL-6 induced epithe-lial-mesenchymal transition in osteosarcoma cell migration and invasion through the STAT3/Snail signaling pathway. On-cology Rep (2017) 38:2647. doi: 10.3892/or.2017.5973 PMC5780017 29048621

